# Erosive and cariogenicity potential of pediatric drugs: study of physicochemical parameters

**DOI:** 10.1186/1472-6831-13-71

**Published:** 2013-12-10

**Authors:** Alidianne Fábia C Xavier, Eline FF Moura, Waldeneide F Azevedo, Fernando F Vieira, Mauro HNG Abreu, Alessandro L Cavalcanti

**Affiliations:** 1 Department of Preventive Dentistry, School of Dentistry, State University of Paraiba, Avenida das Baraunas, S/N, Bodocongo, Campina Grande, PB 58109-970, Brazil; 2 Posgraduate Programa in Public Health, State University of Paraiba, Avenida das Baraunas, S/N, Bodocongo, Campina Grande, PB 58109-970, Brazil; 3 Department of Sanitary and Environmental Engineering, State University of Paraiba, Avenida das Baraunas, S/N, Bodocongo, Campina Grande 58109-970, PB, Brazil; 4 Department of Community and Preventive Dentistry, School of Dentistry, Universidade Federal de Minas Gerais, Av. Antônio Carlos, 6627, Belo Horizonte 31270.901, Brazil

**Keywords:** Dental caries, Drug evaluation, Hydrogen-ion concentration

## Abstract

**Background:**

Pediatric medications may possess a high erosive potential to dental tissues due to the existence of acid components in their formulations. The purpose was to determine the erosive and cariogenic potential of pediatric oral liquid medications through the analysis of their physicochemical properties *in vitro*.

**Methods:**

A total of 59 substances were selected from the drug reference list of the National Health Surveillance Agency (ANVISA), which belong to 11 therapeutic classes, as follows: analgesics, non-steroidal anti-inflammatory, corticosteroids, antihistamines, antitussives, bronchodilators, antibacterials, antiparasitics, antiemetics, anticonvulsants and antipsychotics. Measurement of pH was performed by potentiometry, using a digital pH meter. For the Total Titratable Acidity (TTA) chemical assay, a 0.1 N NaOH standard solution was used, which was titrated until drug pH was neutralized. The Total Soluble Solids Contents (TSSC) quantification was carried out by refractometry using Brix scale and the analysis of Total Sugar Content was performed according to Fehling’s method. In addition, it was analyzed the information contained in the drug inserts with regard to the presence of sucrose and type of acid and sweetener added to the formulations.

**Results:**

All drug classes showed acidic pH, and the lowest mean was found for antipsychotics (2.61 ± 0.08). There was a large variation in the TTA (0.1% - 1.18%) and SST (10.44% - 57.08%) values. High total sugar contents were identified in the antitussives (53.25%) and anticonvulsants (51.75%). As described in the drug inserts, sucrose was added in 47.5% of the formulations, as well as citric acid (39.0%), sodium saccharin (36.4%) and sorbitol (34.8%).

**Conclusion:**

The drugs analyzed herein showed physicochemical characteristics indicative of a cariogenic and erosive potential on dental tissues. Competent bodies’ strategies should be implemented in order to broaden the knowledge of health professionals, drug manufacturers and general consuming public about the risks from the consumption of medicines potentially harmful to dental tissues.

## Background

Oral liquid pharmaceutical dosage forms such as syrups, solutions and suspensions are the therapeutic choice for the treatment of pediatric patients [1]. The use of these liquid preparations, despite being generally for short periods, can be considered as prolonged occurrence [2], especially in patients who suffer from chronic conditions, such as respiratory allergies, asthma and convulsions, or recurrent acute diseases as tonsillitis, otitis, sinusitis and allergic rhinitis [3].

The use of medicines in childhood is high [4], particularly among children under two years old [5], so that the chronic use of medicines to treat asthma and attention-deficit or hyperactivity disorders has been increasing [6]. In Brazil, the prevalence of drug use from three to 12 months of age was estimated to be 65.0%, whereas at 24 months it is 54.7%, with changes observed in the treatment group as a function of age [7].

The fact of the tooth being affected by the use of drugs is based on the assumption that these can reduce saliva flow and buffering capacity [8]. Liquid formulations used for a three-month minimum period are considered a risk factor for increased levels of dental caries [9]. Hence, children who use vitamin C supplements are 4.7 times more likely to develop dental erosion lesions [10].

Studies investigating the physicochemical parameters of pharmaceutical products have provided valuable information on their cariogenic and erosive potential by determining hydrogen potential (pH), titratable acidity, soluble solids content and total sugars [11-17].

### Purpose

This study aimed to evaluate the erosive and cariogenic potential of pediatric oral liquid medications through the analysis of their physicochemical properties *in vitro*.

## Methods

The drugs of this study comprised 94 liquid medicines for children, included in the list of reference drugs by the National Agency for Sanitary Surveillance (ANVISA). As such, this list contains the names of products registered by the Brazilian Ministry of Health, whose effectiveness, safety and quality have been scientifically proven [18].

The sample was composed by medicines commercially available as oral solution or suspension, elixir, syrup and oral suspension powder, which should belong only to the therapeutic classes marketed in three or more types of formulations. Therefore, in accordance with the established parameters, the sample consisted of 59 medicines (62.8% of the universe) categorized in 11 classes and prepared according to their therapeutic indication (Table 1).

**Table 1:** Distribution of medicines according to the therapeutic class, trademark and manufacturer

**Therapeutic class**	**n**	**Trademark and manufacturer**
**Analgesics**	06	Tylenol bebê (Janssen Cilag)^a^, Tylenol criança (Janssen Cilag)^a^, Tylenol gotas (Janssen Cilag)^a^, Novalgina (Sanofi-Aventis)^a^, Novalgina pediátrica (Sanofi-Aventis)^a^, Alivium (Mantecorp)^b^
**Antibacterials**	06	Amoxil BD (Glaxosmithkline)^c^, Zinnat (Glaxosmithkline)^c^, Ceclor (Sigma Pharma)^b^, Keflex (Bagó)^b^, Pen-ve-oral (Eurofarma)^c^, Unasyn (Pfizer)^c^
**Anticonvulsants**	05	Depakene (Abbott)^d^, Tegretol (Novartis Biociência)^b^, Rivotril (Roche)^a^, Gardenal (Sanofi-Aventis)^a^, Trileptal (Novartis Biociência)^b^
**Antiemetics**	03	Digesan (Sanofi-Aventis)^a^, Motilium (Janssen Cilag)^b^, Plasil (Sanofi-Aventis)^b^
**Non-steroidal anti-inflammatory**	04	Profenid (Sanofi-Aventis)^a^, Profenid (Sanofi-Aventis)^d^, Nisulid (Aché)^b^, Cataflam (Novartis Biociência)^b^
**Antihistamines**	07	Zyrtec (Glaxosmithkline)^a^, Zaditen (Novartis Biociência)^a^, Desalex (Mantecorp)^d^, Polaramine (Mantecorp)^a^, Talerc (Aché)^d^, Allegra (Sanofi-Aventis)^b^, Claritin (Mantecorp)^d^
**Antiparasitics**	06	Zentel (Glaxosmithkline)^b^, Flagyl (Sanofi-Aventis)^b^, Pantelmin (Janssen Cilag)^b^, Pyr-pam (UCI Farma)^b^, Thiaben (UCI Farma)^b^, Annita (Famacoquímica)^b^
**Antipsychotics**	03	Amplictil (Sanofi-Aventis)^a^, Haldol (Jansseng Cilag)^a^, Neozine (Sanofi-Aventis)^a^
**Antitussives**	04	Benalet (Johnson & Johnson)^d^, Vibral (Solvay Farma)^a^, Vibral (Solvay Farma)^d^, Cloridrato de Clobutinol (Medley)^d^
**Bronchodilators**	11	Brondilat (Aché)^d^, Fluimucil (Zambon)^d^, Mucolsovan (Boehringer)^a^, Mucolsovan (Boehringer)^d^, Bisolvon (Boehringer)^d^, Berotec (Boehringer)^d^, Aerolin (Glaxosmithkline)^d^, Bricanyl (Astrazeneca)^d^, Transpulmin (Aché)^d^, Xarope Vick (Procter & Gamble)^d^, Xarope Vick Mel (Procter & Gamble)^d^
**Corticosteroids**	04	Celestone (Mantecorp)^e^, Decadron (Aché)^e^, Prelone (Aché)^a^, Predsim (Mantecorp)^a^

^a^Solution, ^b^Suspension, ^c^Oral suspension powder, ^d^Syrup, ^e^Elixir.

Tests were performed in triplicate by a properly calibrated researcher. The Intra-Class Coefficients were determined by comparing the measures of TTA and TSSC of a reference research (PhD in Chemistry) and the calibrated researcher at the Laboratory of Analytical Chemistry, Department of Industrial Chemistry, State University of Paraiba, Campina Grande, PB, Brazil. The Intraclass Correlation Coefficients were both equal to one.

The endogenous pH of each sample was determined by using the digital pH meter HI-253 (Hanna Instruments Brazil Imp. and Exp Ltd., Sao Paulo, SP, Brazil). Initially, samples underwent dilutions in distilled water, so that 10 mL of each medicine were transferred to a 100-mL volumetric flask, and the total volume was reached by adding 90 mL of distilled water. Then 50 mL of the mixture were transferred to a beaker, a glass electrode was immersed therein, and it was proceeded with readings and record of the values obtained [17].

In order to investigate the total titratable acidity (TTA), measure by acidic percentage, it was used a volume of 50 mL of the diluted solution, maintained under constant stirring by using a magnetic stirrer (model 78HW1, Coleman laboratory equipment Com. and Imp. Ltd., Santo Andre, SP, Brazil). The pH meter electrode (HI-253, Brazil Hanna Imp. Ltd. and Exp. Sao Paulo, SP, Brazil) was immersed into this diluted solution at the same time a 0.1 N sodium hydroxide (NaOH) solution was titrated until the pH fell 8.2-8.4, which corresponds to the turning point of phenolphthalein (acid–base indicator that changes color in well-defined pH values). Subsequently, the NaOH volume used was recorded and the acidic percentage of the substance was calculated using the chemical formula described below, whose result had to be adjusted due to the dilution process and expressed as percentage of citric acid [17].

$$\mathrm{Acidity}=\frac{V\times \;\mathrm{Nap}\;\times \;F\times \;\mathrm{meq}‒g\;\times \;100}{\mathrm{Sample}}\;$$

V = NaOH volume; Nap = standard concentration of the KOH (potassium hydroxide) base; F = correction factor; meq-g = milliequivalents *per* gram of citric acid; Sample = drug volume.

The measurements of the Total Soluble Solids Contents (TSSC) or degrees Brix (^o^Bx) were made by refractometry through Brix scale using the Abbe refractometer (PZO-RL1R, Warsaw, Poland). With the aid of a pipette, two to three drops of the medicine were placed on the surface of the measurement prism, avoiding the formation of bubbles in the liquid, since these reduce the contrast of the boundary line that should be located between a light and dark fragment at the intersection point of the reticle. Subsequently, it was necessary to wait a few minutes until the liquid came into thermal equilibrium with the set of prisms. The refractometer was positioned before a natural light source. Also, the incidence angle was adjusted through the activation button and the percentage provided by the equipment was recorded afterwards.

The determination of the total sugar content in the samples was made by the Lane-Eynon method (Fehling) [19]. Initially, it was made the inversion of sucrose (acid hydrolysis) using 25 mL of the medicine, followed by addition of 5 ml of hydrochloric acid (HCl) and 75 mL of distilled water. The diluted solution contained in a flask was heated to 70°C and remained in water-bath for ten minutes, in order to obtain glucose and fructose molecules. Then, the mixture was cooled in water and had its pH neutralized by titrating with 30.0% NaOH. The neutral solution obtained was then transferred to a 25-mL burette. In parallel, 5 ml of solution A and 5 ml of solution B of the Fehling reagents were mixed in a porcelain capsule; 40 ml of distilled water were added and the mixture underwent boiling for four minutes. It was used the content of the burette as titrant agent and the emergence of a red brick-like precipitate as an indicator of the turning point. The end point of the reaction was indicated by methylene blue, which was reduced to a colorless form by slight excess of reducing sugar.

The volume required was recorded and the percentage of total sugars present in the sample was calculated according to the following equation:

$$\mathrm{Total}\;\mathrm{sugars}\;\left(\%\right)=\frac{F_{\mathrm{EO}}\times \;\mathrm{DILUTION}\;\times \;100}{V_{\mathrm{TITRATION}}}$$

F_EQ_ = Equivalence factor; V_TITRATION_ = Titration volume required.

In addition to the *in vitro* analysis, information concerning the type of acid and sweetener contained in the formulations were gathered from the medicine package inserts.

Data were recorded on study-specific charts and organized with the aid of the *Statistical Package for Social Sciences* (SPSS) version 18 and presented by means of descriptive statistics (mean, standard deviation, minimum and maximum values).

## Results

The pH values were in the range 2.43 (Claritin) to 7.48 (Predsim); the mean was 5.01 (± 1.47 SD) and 55.9% of the medicines were found to show a pH below 5.5. The analysis of the mean of each group highlighted the fact that all samples exhibited an acidic pH, and the lowest mean value (2.61) was found for antipsychotics.

The TTA analysis by treatment group showed a variation in the means obtained (0.10% to 1.17%). Individually, the occurrence of high acidity for the medicines: Vibral solution (1.23%); Ceclor (1.57%); Amplictil (1.54%) and Neozine (1.56%) was observed (Table 2).

**Table 2:** Distribution of medicines according to the therapeutic class, mean, standard deviation (SD), median (Md), and minimum (Min) and maximum (Max) values of pH and TTA

**Therapeutic class**	**pH**				**TTA**			
	**Mean (SD)**	**Md**	**Min**	**Max**	**Mean (SD)**	**Md**	**Min**	**Max**
Analgesics	4,8 (1.4)	4.1	3.5	6.7	0.2 (0.1)	0.3	0.02	0.4
Antibacterials	6.0 (0.9)	6.1	4.5	7.3	0.5 (0.5)	0.3	0.07	1.5
Anticonvulsants	4.9 (1.9)	3.9	2.8	7.4	0.2 (0.1)	0.1	0.05	0.4
Antiemetics	4.6 (1.6)	4.1	3.3	6.4	0.1 (0.0)	0.1	0.07	0.1
Non-steroidal anti-inflammatory	5.6 (1.1)	6.0	4.0	6.5	0.1 (0.0)	0.1	0.05	0.1
Antihistamines	5.3 (1.5)	5.6	2.4	7.2	0.2 (0.2)	0.1	0.03	0.7
Antiparasitics	5.3 (1.1)	5.3	3.8	6.6	0.1 (0.0)	0.1	0.06	0.2
Antipsychotics	2.6 (0.0)	2.5	2.5	2.7	1.1 (0.6)	1.5	0.43	1.5
Antitussives	4.3 (1.0)	4.5	3.0	5.3	0.3 (0.5)	0.1	0.06	1.2
Bronchodilators	4.9 (1.0)	4.8	3.6	6.5	0.2 (0.1)	0.2	0.04	0.5
Corticosteroids	5.1 (2.5)	5.1	2.8	7.4	0.1 (0.1)	0.0	0.04	0.3

Evaluation of medicine package inserts demonstrated that although citric acid has been often added (39.0%) in the composition of the formulations, other acids such as benzoic, sorbic, acetic, hydrochloric, tartaric, estereatic, ascorbic and lactic acids are also incorporated.

Total Soluble Solids Contents were measured in all medicines, and the lowest contents were found for Aerolin (1.42%); Digesan (1.0%); and Haldol (1.0%); while the highest contents were identified in Tylenol drops (69.67%) and Depakene (65.33%). The mean percentage of total sugars identified was 30.9% (± 15.5); the minimum value of 7.31% was estimated for the antibiotic Keflex, while Vibral solution, Pen-v-oral and Unasyn reached the mark of 54.8%. The mean, minimum and maximum values for TSSC and sugar content are summarized in Table 3.

**Table 3:** Distribution of medicines according to the therapeutic class and mean, standard deviation (SD), median (Md), and minimum (Min) and maximum (Max) values of TSSC and sugar content

**Therapeutic class**	**TSSC**				**Sugar content**			
	**Mean (SD)**	**Md**	**Min**	**Max**	**Mean (SD)**	**Md**	**Min**	**Max**
Analgesics	55.6 (15.8)	62.0	25.5	69.6	33.7 (0.8)	33.7	32.9	34.6
Antibacterials	57.0 (11.9)	61.3	33.3	64.9	30.7 (21.3)	30.0	7.31	54.8
Anticonvulsants	41.1 (20.2)	43.4	20.2	65.3	51.7 (5.2)	54.8	45.6	54.8
Antiemetics^a^	10.4 (15.4)	2.0	1.0	28.2	-	-	-	-
Non-steroidal anti-inflammatory	31.5 (19.7)	26.5	14.8	58.2	40.0 (16.5)	40.0	28.4	51.7
Antihistamines	48.4 (9.9)	50.5	29.1	60.7	27.7 (17.6)	22.9	13.4	30.3
Antiparasitics	26.5 (18.0)	24.3	3.5	58.5	18.2 (7.6)	18.3	10.1	25.8
Antipsychotics	31.4 (26.4)	46.3	1.0	47.0	21.9 (1.4)	21.9	20.9	22.9
Antitussives	45.3 (13.9)	46.6	29.5	58.5	53.2 (2.1)	53.2	51.7	54.8
Bronchodilators	31.1 (20.0)	33.0	1.4	56.0	32.9 (13.6)	30.4	19.2	51.7
Corticosteroids	33.2 (12.3)	32.1	20.0	48.5	25.1 (4.3)	27.7	20.1	27.7

^a^Absence of sugar in the composition.

According to the analysis of the inserts, sucrose was found in 47.5% of the medicines, and the antiemetics drug class was the only one devoid of sugar products (Table 4). With regard to the samples lacking sucrose, it was found that saccharin and sorbitol were the artificial substitutes of choice, since these were added to about 37.0% and 35.0% of the medicines, respectively. Other sweeteners like sodium cyclamate, aspartame, acesulfame, xylitol and sucralose were also used, either alone (37.3%) or associated. The combination of two, three and four sweeteners was found in 25.4%, 5.1% and 1.7% of the cases, respectively.

**Table 4:** Distribution of medicines according to the therapeutic class and presence of sucrose as described in the inserts

**Presence of Sucrose**						
	**Yes**		**No**		**Total**	
**Therapeutic class**	n	%	n	%	n	%
Analgesics	03	50.0	03	50.0	06	10.2
Antibacterials	06	100.0	0	0.0	06	10.2
Anticonvulsants	01	20.0	04	80.0	05	8.5
Antiemetics	0	0.0	03	100.0	03	5.1
Non-steroidal anti-inflammatory	02	50.0	02	50.0	04	6.8
Antihistamines	04	57.1	03	42.9	07	11.9
Antiparasitics	05	83.3	01	16.7	06	10.2
Antipsychotics	02	66.7	01	33.3	03	5.1
Antitussives	02	50.0	02	50.0	04	6.8
Bronchodilators	04	36.4	07	63.6	11	11.9
Corticosteroids	01	25.0	03	75.0	04	6.8
**Total**	**30**	**50.8**	**29**	**49.2**	59	100.0

n = number of samples.

## Discussion

The wide range of products available in the national therapeutic arsenal ranks Brazil among the countries that have a strong consumer market. Nevertheless, there is consensus among health professionals that liquid dosage forms, particularly oral solutions and suspensions, are the most appropriate ones for use in pediatrics, because in addition to facilitating the administration and contributing to patient compliance to therapy, these preparations have great flexibility, allowing to adjust the doses given during treatment simply and rapidly, depending on the pathology and child development [1,20].

A number of investigations on liquid medicines belonging to different therapeutic classes have been conducted since the last decade, in order to produce reliable information on the physicochemical profile of medicines used by children, both in international [11,13] and Brazilian studies [12,14,16,17].

The analysis of pH is an important variable involved in the process of dental erosion [21]. Current literature shows that there is a range from 2.5 [17] to 6.9 [15] for pH values of medicines. Specifically, according to the findings reported herein most samples were found to show an acidic pH below 5.5, which points to their sub-saturated condition in relation to the tooth hydroxyapatite. Despite the shortcomings of an *in vitro* study, it is speculated that the presence of a lower pH in the formulations is characterized as a predictor of dental erosion. Some users manual gives mostly the advice to take the medicine with water. If family compliance with this advice, the risk of dental erosion could be lower.

Antipsychotics and antitussives were the therapeutic classes showing the lowest pH values. This feature should be observed carefully, since the therapy using antipsychotic drugs requires, in many cases, ingestion of the solution several times a day and for an indefinite period of time. Moreover, it is knowingly reported in the literature that cough is a common condition occurring in children, so that antitussive drugs are widely used, sometimes without proper medical guidance.

The acidic pH prevents liquid medicines from being contaminated with microorganisms [22]. Additionally, it can be mentioned the fact that this characteristic is directly related to factors such as chemical stability and biocompatibility of the active agent [23].

Pharmacologically, it is clear that the establishment of inappropriate pH values during the drug formulation process may either favor the decomposition of the active ingredient or harm its therapeutic activity [24,25]. From the perspective of dentistry, it is configured an alarming and conflicting situation as on one hand it is recognized the importance of the medicine as an irreplaceable tool for restoring and maintaining children’s health status, on the other hand it is perceived the possibility of organ involvement by dental caries and dental erosion.

The acidic contents are added to medicines as they act as buffering agents, responsible for maintaining chemical stability, controlling tonicity, ensuring the physiological compatibility and improving flavor, which make medicines more palatable to the child [13].

In this study, citric acid was found in the most part of the medicines tested, corroborating data from previous studies [13,16]. This acid is a potent erosive agent, because it has the ability to chelate calcium, increasing dental enamel dissolution rates upon the acid challenges that are imposed^25^. The presence of benzoic, tartaric and hydrochloric acids was observed in a number of medicines, confirming previous findings [16].

The estimation of total titratable acidity establishes an indirect measure of the amount of saliva buffer required to provide the medicine with a neutral pH. Saliva buffering capacity is directly associated to the presence of bicarbonate in the salivary flow. During the resting period, the concentration of this compound is decreased, though there are peptides, amino acids and phosphates that are also involved in the buffering mechanism. Nevertheless, after administration, acidic medicines stimulate saliva flow and release of greater amounts of bicarbonate [11].

Therefore, it was investigated the percentage of acid of the formulations by titrimetric analysis, in which a known standard NaOH solution was used. It is emphasized that we could not perform the tests in the medicine whose active ingredient was ibuprofen. This might be explained by the fact that such drug is practically insoluble in water. For the other samples, it was found a wide spectrum of variation, possibly due to the peculiarities of the active ingredients. Studies analyzing antitussive medicines with similar methodologies have shown TTA means of 0.18% [14] and 0.29% [17]. These results are lower than 0.39% obtained in this study for the antitussive samples.

The test for detection of soluble solids in medicines used refractometry as the method of choice, because despite some drawbacks (such as loss of sensitivity and temperature dependency) [26] it has been used in studies assessing physicochemical parameters of oral liquid medicines [14,17]. In this study, the soluble solids means differed among the samples, with lower values found for the antiemetics class, which did not have sugar in their composition. High percentages were found for antibiotics and analgesics, which supposedly have the potential to develop dental caries.

Sugars are multifunctional ingredients added to drug formulations due to the unpleasant taste of many active constituents. In addition, sucrose is easily processed and available in different dry particle sizes, chemically and physically stable, acting as an oxidant and solvent, and providing viscosity to the medicine. It is not hygroscopic and is less costly, which influences the final product price [27].

With regards to the use of medicines containing sugary vehicles, it was found that approximately half of the samples tested had sugar in their composition, reaching a mean rate of 53.2% for antitussives, which is slightly higher than the 48.0% rate identified in a recent study [17]. However, higher sugar contents were observed in other studies with percentages ranging from 65.0% [15] to 86.9% [17].

The presence of sucrose in the medicines is noteworthy, given that all antipsychotics had such disaccharide contained in their formulations. Moreover, it was observed that the dosage description of Neozine explicitly recommends its dilution in “sugared-water”. Thus, children using this substance may have a harmful cumulative effect that might be manifested as carious and erosive lesions on the tooth surface, taking into account that such medicine has a pH value below that for enamel dissolution, high acidity and sucrose in its formulation.

As aforementioned, liquid medicines usually have unpleasant taste, so it has been necessary to include various sweeteners in the same product to overcome this. In this research, in addition to sucrose, the sweeteners commonly found were sodium saccharin, sorbitol and sodium cyclamate. Hypothetically, it is assumed that the choice for one or another type of sweetener, or even the combination of many, as seen in 25.0% of the medicines studied, is not only due to the given degree of sweetness, but also to its compatibility with the active ingredients.

Considering all the afore-listed features, competent bodies’ strategies should be implemented in order to broaden the knowledge of health professionals, drug manufacturers and general consuming public about the risks from the consumption of medicines potentially harmful to dental tissues.

Mainly owing to the fact that regulatory maneuvers are now being instituted in other countries, the *European Medicines Agency* established in 2007 a set of measures involving specific regulation for drug registration and incentives for clinical research and development of medicines for pediatric use [28].

## Conclusions

•Most medicines were found to show acidic pH values lower than that needed for tooth enamel dissolution;

•Anticonvulsants and antitussives were considered potential risk factors for the development of dental caries, as they presented significant TSSC and total sugars in their composition.

## Competing interests

The authors declare that they have no competing interests.

## Authors’ contributions

AFCX and EFFM participated in the design of the study, carried out the data collection, conducted statistical analyses and helped draft the manuscript. ALC and MHNGA conceived and designed the study, coordinated and carried out statistical analyses and drafted the manuscript. FFV and WFA participated in the design of the study and carried out the data collection. All authors contributed to the writing of the manuscript and critically reviewed the final version. All authors read and approved the final manuscript.

## Pre-publication history

The pre-publication history for this paper can be accessed here:

http://www.biomedcentral.com/1472-6831/13/71/prepub
